# A Three-Enzyme-System to Degrade Curcumin to Natural Vanillin

**DOI:** 10.3390/molecules20046640

**Published:** 2015-04-14

**Authors:** Vida Esparan, Ulrich Krings, Marlene Struch, Ralf G. Berger

**Affiliations:** Institut für Lebensmittelchemie im Zentrum Angewandte Chemie, Gottfried Wilhelm Leibniz Universität Hannover, Callinstraße 5, D-30167 Hannover, Germany; E-Mails: krings@lci.uni-hannover.de (U.K.); marlene.struch@lci.uni-hannover.de (M.S.); rg.berger@lci.uni-hannover.de (R.G.B.)

**Keywords:** vanillin, curcumin, lipase, laccase, esterase

## Abstract

The symmetrical structure of curcumin includes two 4-hydroxy-3-methoxyphenyl substructures. Laccase catalyzed formation of a phenol radical, radical migration and oxygen insertion at the benzylic positions can result in the formation of vanillin. As vanillin itself is a preferred phenolic substrate of laccases, the formation of vanillin oligomers and polymers is inevitable, once vanillin becomes liberated. To decelerate the oligomerization, one of the phenolic hydroxyl groups was protected via acetylation. Monoacetyl curcumin with an approximate molar yield of 49% was the major acetylation product, when a lipase from *Candida antarctica* (CAL) was used. In the second step, monoacetyl curcumin was incubated with purified laccases of various basidiomycete fungi in a biphasic system (diethyl ether/aqueous buffer). A laccase from *Funalia trogii* (LccFtr) resulted in a high conversion (46% molar yield of curcumin monoacetate) to vanillin acetate. The non-protected vanillin moiety reacted to a mixture of higher molecular products. In the third step, the protecting group was removed from vanillin acetate using a feruloyl esterase from *Pleurotus eryngii* (PeFaeA) (68% molar yield). Alignment of the amino acid sequences indicated that high potential laccases performed better in this mediator and cofactor-free reaction.

## 1. Introduction

Flavors and fragrances originate from traditional extraction or distillation of plant and animal sources or from chemosynthesis, but the quality and the stability of the natural supplies are sometimes limited. Effective law in Europe (EG 1334/2008) and in the U.S. (Code of Federal Regulation, Title 21) defines flavors with the preferred label ‘natural’ as compounds obtained by physical, enzymatic or microbiological processes. As this disqualifies chemical synthesis, biotechnological approaches have moved into focus [1]. Biocatalysis represents an economic alternative using either intact cells or isolated enzymes, such as laccases [2], often resulting in the formation of products difficult to obtain by conventional chemical means. Enzymes possess a long history of safe use in producing fermented foods. They accelerate just one reaction without the ballast of an ongoing metabolism of a whole cell. Technically well manageable, many technical enzymes have become amenable through recombinant hosts expressing the target enzyme in good yield and purity [3].

With an annual consumption of an estimated 15,000 tons, vanillin (4-hydroxy-3-methoxybenzaldehyde) is one of the most widely-used flavor compounds in baked goods, chocolates, dairy products, perfumes and even pharmaceuticals. Only 0.2% of the total demand is provided from vanilla beans, while the rest is supplied by chemical synthesis and a ferulic acid-based bioprocess. Natural vanilla flavor is a complex of many components, but the aroma is largely determined by vanillin. Because of the scarcity and high cost of natural vanilla extract, there has been a continuing interest in its biotechnological production. There are different possibilities for the production of natural vanillin, such as biotransformation of caffeic acid and veratryl aldehyde, or the fermentation of natural substrates, such as ferulic acid, eugenol, isoeugenol, coniferyl alcohol, vanillin alcohol and stilbene, by bacteria and fungi, such as *Pseudomonas fluorescens*, *Escherichia coli*, *Amycolatopsis* sp., *Streptomyces setonii*, *Pycnoporus cinnabarinus* or *Aspergillus niger* [4,5,6,7]. Ferulic acid is available in abundance in plant cell walls and has become the most popular precursor substrate. The increasing price of ferulic acid has stimulated the search for other natural precursor molecules to obtain vanillin naturally.

Curcumin occurs in turmeric (*Curcuma longa*) rhizome powder, a common ingredient of curry spice, in concentrations of up to 3%. It is a food colorant (E 100) and was claimed to exhibit numerous wide biological functions, although the bioavailability of curcumin is low [8]. The two phenolic rings at the molecule ends are connected by two *α,β*-unsaturated carbonyl moieties. A hypothetical cleavage at the benzylic position would yield two moles of vanillin from one curcumin molecule. Because of physico-chemical and structural features similar to lignin-related compounds, it was supposed that lignin-degrading microorganism may also be able to degrade curcumin. Previously, *Rhodococcus* strains have been reported as promising candidates, which degraded curcumin to (*E*)-6-(4'-hydroxy-3'-methoxyphenyl)-2,4-dioxo-5-hexenal, feruloylmethane, ferulic acid and vanillin [9]. The aim of the present study was to develop an enzyme-based route starting with curcumin and resulting in vanillin as the most abundant reaction product.

## 2. Results and Discussion

For 20 years, the degradation of natural ferulic acid to vanillin using an optimized bacterial strain (*Amycolatopsis* family) has been one of a few successful large-scale processes using whole cell cultures for the production of a natural flavor compound [10]. Alternative precursors and routes to natural vanillin have been intensively researched, including the symmetric cleavage of curcumin. Its autoxidative degradation at physiological conditions led to the incorporation of oxygen into a curcumin radical resulting in a bi-substituted bicyclopentadione structure, while vanillin, ferulic acid and feruloylmethane occurred as minor degradation products [11]. A more concerted enzymatic cleavage at both benzylic positions using either a whole cell system or an oxidoreductase could be envisaged. One curcumin molecule would result in the formation of two molecules of vanillin, and a cofactor-independent enzyme would be most preferred.

**Figure 1:**
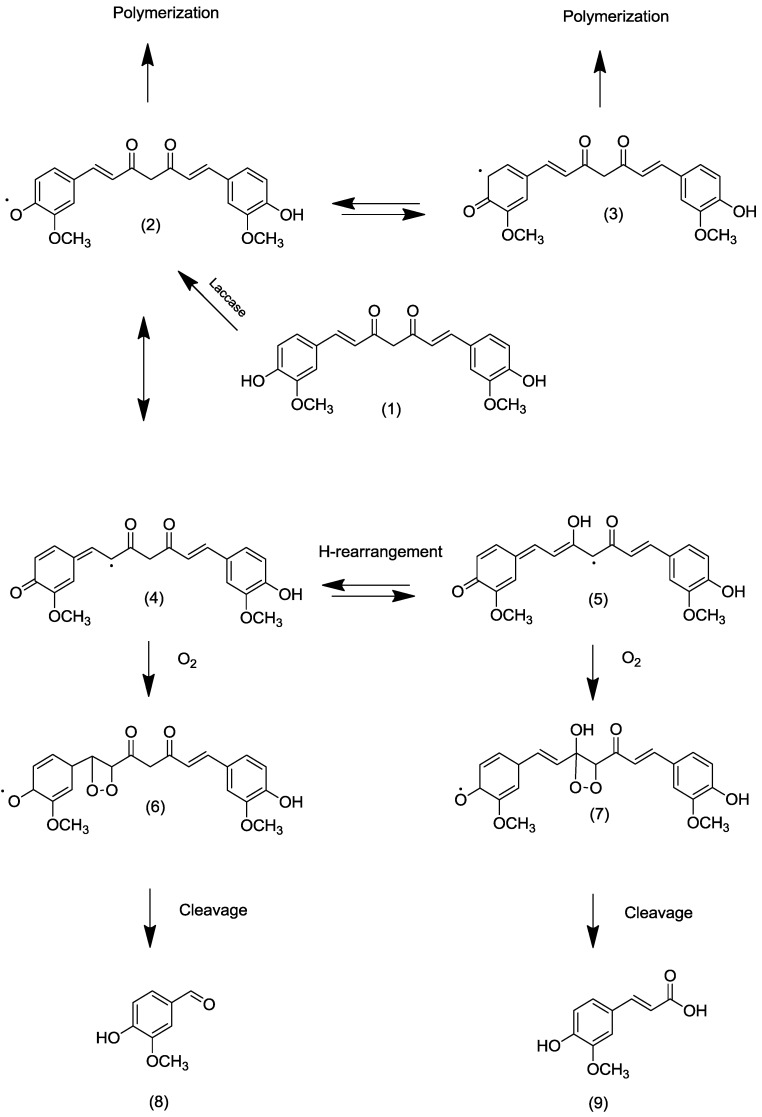
Hypothetical pathway of laccase-catalyzed biotransformation of curcumin. (1) Curcumin; (2) O-centered curcumin radical; (3) C-centered curcumin radical; (4,5) C-centered radicals in the alkenyl chain of curcumin; (6,7) intermediate 1,2-endoperoxides of the curcumin radical; (8) Vanillin; (9) Ferulic acid.

Abstraction of a hydrogen from a phenol with subsequent oxidation of the substrate is the domain of fungal laccases. These multi-copper oxidases (E.C.1.10.3.2) form resonance-stabilized phenol radicals directly or by the aid of mediators, such as caffeic acid, vanillin (natural) or 2,2'-azino-bis(3-ethylbenzthiazoline-6-sulphonic acid) (ABTS, non-natural), and reduce molecular oxygen to water at the same time [12]. The cofactor and mediator-free direct incubation of curcumin with various laccases of different redox potentials resulted in an immediate degradation of curcumin (as measured by HPLC) and visible formation of a buffer-insoluble precipitate. However, the targeted degradation products, mainly vanillin (and ferulic acid), were found in traces only. Being phenols themselves, they were preferred substrates for the laccases, resulting in oligo-/polymerization of the intermediate monomers. To arrive at the intended benzylic cleavage, a less reactive substrate was required. A hypothetical mechanism would imply the delocalization of the unpaired electron of the phenoxy radical into the side chain and, after tautomerization and insertion of molecular oxygen, the generation of respective 1,2-endoperoxides; these, in turn, are well known to decay into two carbonyl moieties (Figure 1) [13].

### 2.1. Acetylation of Curcumin

#### 2.1.1. By a Chemical Route

To reduce the suitability of curcumin as a laccase substrate, it was aspired to achieve the acetylation of at least one of the phenolic hydroxyl groups of the molecule. Chemical formation of acetyl curcumins yielded two pairs of peaks with identical molecular masses, corresponding to monoacetyl, *m/z* 409, ESI(−) with 50%, and diacetyl curcumins, *m/z* 451, ESI(−) 50% by mass of total reaction products. The respective major peak of each pair was assigned to the phenolic acetyl/diacetyl ester with 93% by mass, whereas the minor peaks (7% by mass) were assigned to the acetylated hydroxyl group of the tautomeric form of curcumin. The reaction mixture was partially purified by means of preparative TLC. A curcumin-free mixture after purification, which was composed of around 90% monoacetyl curcumin and 10% of diacetyl curcumin, was used as the substrate for the following cleavage by three fungal laccases possessing different redox potentials.

#### 2.1.2. By an Enzymatic Route

For the production of ‘natural’ vanillin, an enzyme-based formation of acetyl curcumin is mandatory. Therefore, reverse hydrolysis was adapted for the acetylation of curcumin in organic medium. A number of commercial lipases are available for acetate synthesis. CAL, a lipase from *Candida antarctica*, was frequently used, and vinyl acetate served as the acyl donor, thus forcing the equilibrium to the product side by tautomerization of the liberated vinyl alcohol. Yields were semi-quantified by LC-MS, but remained unsatisfactory. Reaction solvent, temperature, time and the molar ratio of the reactants were varied. A maximum yield of approximately 49% was eventually achieved using geranyl acetate as the acyl donor and a molar ratio of 1:50 of curcumin to acetyl donor. The yield of monoacetyl curcumin increased continuously over time until day three and decreased again thereafter. Even after a long time of incubation, only traces of diacetyl curcumin were detected. It may be speculated that the large and inflexible curcumin molecule does not fit well into the deep substrate-binding site of this lipase. The larger monoacetyl curcumin fitted even less well, thereby preventing diacetylation.

### 2.2. Transformation of Acetyl Curcumin by Laccases

The commercial laccase LccAbi of *A. bisporus* and two laccases, recovered and purified from supernatants of cultivated fungal strains, LccMgi (*M. giganteus*) and LccFtr (*F. trogii*), were compared (Table 1) [14]. Iso-active (1.19 U·mL^−1^ adjusted against ABTS as a substrate) laccase preparations in buffered aqueous solution were added to acetyl curcumins in different solvent systems; these were monophasic organic solvents, monophasic water/water miscible organic solvents and biphasic systems composed of water/water immiscible organic solvents 

**Table 1:** Characteristics of laccases used for the degradation of acetyl curcumin.

Laccase	Origin	Redox Potential ^a^	pI	pH Optimum ^b^	Temperature Optimum ^b^ (°C)
LccAbi	*A. bisporus*	Middle (0.47–0.71 V)	3.5	4.5–5	30–40
LccMgi	*M. giganteus*	High (0.73–0.78 V)	3.1	5–5.5	30–40
LccFtr	*F. trogii*	High (0.73–0.78 V)	3.8	4.5–5	30–40

^a^ According to the literature; ^b^ according to 2,2'-azino-bis(3-ethylbenzthiazoline-6-sulphonic acid) (ABTS) enzyme activity.

The best reaction conditions were found to be a biphasic system consisting of water/diethyl ether. Samples were taken after 20 h and analyzed by LC-MS for substrate transformation and possible polymerization products and GC-MS for volatile degradation products. In all incubations in the presence of a laccase, as well as in the controls, diacetyl curcumin remained stable and did not change concentration over time. Monoacetyl curcumin concentration, in contrast, declined with time depending on the laccase added. For LccAbi, no distinct degradation occurred, whereas for LccMgi, a 15% and for LccFtr a 46% decline of the monoacetyl curcumin concentration were observed. During the reaction, the yellow bright reaction solution did not show visible alteration with LccAbi, but turned into yellow/brownish with little precipitation with LccMgi and became cloudy with LccFtr. Several molecular masses in the *m/z* range >600 Da were detected over a broad retention time window in the LC-MS chromatograms of these samples, indicating the formation of oligomer phenols, but the structural elucidation of these was no the aim of this study. GC-MS analysis of the volatile reaction products showed just one major product, acetyl vanillin. This was expected, because other possible degradation products, such as vanillin or ferulic acid, were good substrates for the laccases and polymerized *in situ*, as discussed above. The highest concentration of acetyl vanillin was analyzed for LccFtr, which agreed with the rapid degradation of monoacetyl curcumin (Table 2).

**Table 2:** Yield of vanillin acetate after cleavage of monoacetyl curcumin in a biphasic system: 2 mL of 0.5 mM monoacetyl curcumin in diethyl ether and 2 mL aqueous buffer of laccases (each set to 1.19 U·mL^−1^) under continuous mixing for 20 h at 20 °C.

Laccase	Vanillin Acetate (mg·L^−1^)	Vanillin Acetate (mM)	Molar Product Yield (%) *
LccAbi	6.4	0.032	6.4
LccMgi	15.1	0.078	15.6
LccFtr	45.02	0.23	46

* Calculated according to the concentration of the actual precursor, monoacetyl curcumin.

### 2.3. Alignment of the Laccases

**Figure 2:**
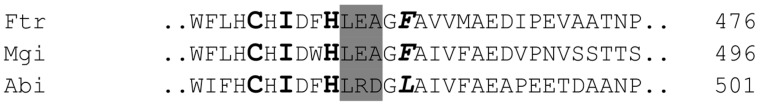
Partial amino acid alignment of LccFtr (*Funalia trogii*), LccMgi (*Meripilus giganteus*) and LccAbi (*Agaricus bisporus*). Bold letters show three out of four invariable T1 copper ligands, bold and italic letters the variable axial ligand and letters highlighted in grey a characteristic tripeptide of the binding site of T1 copper indicative of the redox potential of the respective laccases.

Different amino acid substituents near the substrate binding site and copper T1 coordination of laccases result in different potentials of the redox centers, thus affecting the catalytic properties of bacterial [15] and fungal laccases [16]. To better explain the observed differences in reactivity, the respective parts of amino acid sequences were aligned (Figure 2). According to previous studies, the T1 copper shows a trigonal bipyramidal coordination with three highly-conserved trigonal ligands (H,C,H) and two weakly coordinated ligands in axial position, of which one is invariable Ile, whereas the second is variable. There is a modest correlation between this axial ligand and the redox potential of T1 copper, with Phe consistently producing high, Leu middle and Met low potentials. An adjacent tripeptide (LEA in terms of high potential laccases), which is part of the T1 pocket, also serving as part of the substrate-binding pocket, is indicative of the respective redox potential of laccases, as well [16,17]. The comparison of the sequences of the laccases from Ftr, Mgi and Abi with literature data showed that with Phe, the axial ligand located in position 460 for Laccase Ftr and 480 for laccase Mgi; both had to be classified as high redox potential-type enzymes and LccAbi, having leucine in the axial position 485, as a midrange potential enzyme. The different oxidation rates of the laccases with monoacetyl curcumin as the substrate may be explained by these differences in the amino acid sequences and, consequently, redox potential. High potential laccases appear to be more suitable for the cleavage of C = C bonds in the side chain of curcumin.

### 2.4. Enzymatic Deacetylation of Acetyl Vanillin

Vanillin acetate was obtained as the major volatile compound of the laccase-catalyzed degradation of monoacetyl curcumin. To achieve the enzyme-catalyzed deacetylation of acetyl vanillin, three different esterases were compared (Table 3). At an optimum reaction temperature of 37 °C, the esterase PeFaeA deacetylated 68% of acetyl vanillin to vanillin after five hours of incubation, as calculated by external standard-based GC-flame ionization detection (FID) and GC-MS analyses.

**Table 3:** Yield of vanillin after deacetylation of vanillin acetate in a biphasic system: 2 mL of 1 mM vanillin acetate in diethyl ether and hexane (5:95) and 2 mL aqueous buffer of esterases (each set to 1 U·mL^−1^) under continuous mixing for 5 h at 37 °C.

Esterase	Vanillin (mg·L^−1^) *	Vanillin (mM)	Molar Product Yield (%)
UmChlE	0	0	0
Porcine liver	75.5	0.50	50
PeFaeA	103	0.68	68

* Calculated according to the external standard (3,4-dimethoxybenzaldehyde).

**Figure 3:**
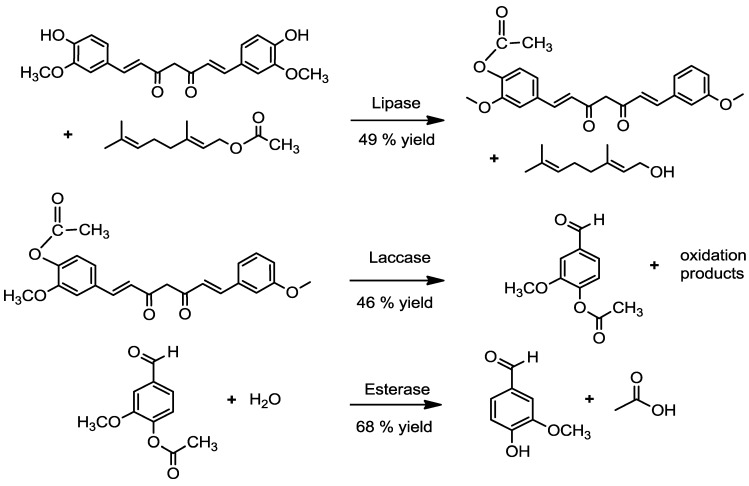
Three-step enzymatic bioconversion of curcumin to natural vanillin.

In summary, many different possibilities for the biotechnological production of vanillin have been investigated in the past. Most processes were primarily affected by the high chemical reactivity and toxicity of vanillin. Thus, three-step enzymatic reactions are a novel approach to produce natural vanillin from curcumin (Figure 3).

## 3. Experimental Section

### 3.1. Materials

All chemicals were analytical grade. Curcumin (>90%, natural) was purchased by Roth (Karlsruhe, Germany). 3,4-dimethoxybenzaldehyde and geranyl acetate from Sigma-Aldrich (Taufkirchen, Germany). 2,2'-Azino-bis (3-ethylbenzthiazoline-6-sulfonic acid) diammonium salt (ABTS) and *p*-nitrophenyl butanoate were obtained from ICN Biochemicals (Muenchen, Germany). Diethyl ether, ethyl acetate, toluene and n-pentane were from Karl Roth (Karlsruhe, Germany), and solvents (all MS grade) used for HPLC-MS were from Carlo Erba Reactifs (Peypin, France).

### 3.2. Enzymes

Immobilized lipase (triacylglycerol hydrolase, EC 3.1.1.3 (Novozyme_435, 5000 U·g^−1^)) from *Candida antarctica* and laccase from *Agaricus bisporus* (6.8 U·mg^−1^) were from Sigma-Aldrich (Taufkirchen, Germany), and esterase from porcine liver (lyophilisate, 15 U·mg^−1^) was from Sigma Aldrich (Taufkirchen, Germany). Recombinant feruloyl esterase from *Pleurotus eryngii* (PeFaeA) and chlorogenic acid esterase from *Ustilago maydis* (UmChlE) were selected from our own stocks. Two further laccases were isolated from fungal culture supernatants, as described below. The strains were purchased from the Centraalbureau voor Schimmelcultures (*Meripilus giganteus* CBS 561.86) and from the German Collection of Microorganisms and Cell Cultures (*Funalia trogii*, DSMZ), respectively.

### 3.3. Cultivation of Fungi

The culture supernatant of *M. giganteus* was provided according to the paper of Schmidt *et al*. [18]. Submerged pre-culture of *F. trogii* was inoculated with the same structure of *M. giganteus*,except that for the main cultures, the expression of laccases was induced either by the addition of three grams per 100 mL^−1^ wheat bran and CuSO_4_ (300 µM final concentration) to the culture medium of *F. trogii* or 300 µM CuSO_4_ solely in the case of *M. giganteus*. At the time of maximum laccase activity (ABTS activity, pH 3.0), cultivation was stopped and the culture supernatant harvested and stored at −20 °C, unless used immediately for laccase isolation and purification.

### 3.4. Laccase Isolation and Purification

The laccase from *M. giganteus* was isolated according to the protocol of Schmidt *et al.* [18]. In brief, the supernatant was frozen at −20 °C, thawed and centrifuged at 25,000× *g*. After filtration using a 0.45-µM polyester filter (CHROMAFIL PET-45/25, Macherey-Nagel, Dueren, Germany) and concentration using an ultra-filtration module (30-kDa cut-off, PES, Sartorius, Goettingen, Germany), the laccase was purified using fast protein liquid chromatography (Biologic Duoflow TM, Bio-Rad, Hercules, CA, USA) at 4 °C. First, a weak anion exchange column was applied (HiPrep 16/10 DEAE, 16 × 100 mm fast flow, GE Healthcare, Munich, Germany). Concentrated laccase fractions were submitted to a second purification using size exclusion chromatography (Superdex 75 10/300 GL column, GE Healthcare, Munich, Germany). Active fractions were pooled and adjusted to the activity required.

Laccase from *F. trogii* was purified as follows: The culture supernatant was frozen at −20 °C, thawed and centrifuged at 5000× *g* at 4 °C for 15 min. The supernatant was filtered (0.45 µM, Chromafil Pet-45/25, Dueren, Germany), concentrated using an ultrafiltration module (30-kDa cut-off, PES, Sartorius, Goettingen, Germany) and subjected to fast protein liquid chromatography (Biologic Duoflow TM, Bio-Rad, Hercules, United States) at 4 °C. Twenty five milliliters of concentrated solution were purified on a HiPrep 16/10 DEAE, 16 × 100 mm fast flow column with a flow rate of 3 mL·min^−1^ (GE Healthcare, Munich, Germany) with 20 mL running Buffer A (50 mM, potassium phosphate, pH 6.5) and eluted with 5% Buffer B (50 mM potassium phosphate, pH 6.5 + 1 M NaCl). Purification was controlled using SDS-PAGE electrophoresis.

SDS-PAGE was performed using 12% (w/v) polyacrylamide gels. Samples were diluted in native loading buffer (0.05 M Tris/HCl pH 6.8, 0.1% bromophenol blue, 10% glycerol, 2% SDS) and applied to electrophoresis. Proteins were stained with ready-to-use Instant Blue solution (0.1%, Expedeon, Cambridge, UK). Laccase activity staining was performed directly on the gel using ABTS (5 mM in 100 mM sodium phosphate buffer pH 4.5).

### 3.5. Analysis of Amino Acid Sequence

The identity of the purified laccases, as well as the sequence of lccAbi were deduced from the amino acid sequence of tryptic peptides of cut out protein bands from SDS gel electrophoresis. De-staining and tryptic digestions of the respective protein bands were carried out as described elsewhere [19]. Tryptic peptides were analyzed by means of nano-LC EASY-nLC II (Bruker Daltronik, Bremen, Germany) equipped with a 20-mm pre-column (C18-A1 3PCS; ThermoFisher Scientific, Dreieich, Germany) and a capillary column (0.1 mm × 150 mm) packed with Magic C18 AQ (3-mm particle size, 200-Å pore size; Michrom Bioresources, Inc., Auburn, CA, USA) eluted by a linear gradient (300 nL·min^−1^) of water and acetonitrile, each with 0:1% formic acid v/v from 95% water to 95% acetonitrile within 25 min and held for 15 min. The amino acid sequences elucidated were subjected to protein database (NCBI, Mascot search algorithm). Sequences were aligned using the ClustalW2 multiple sequence alignment database.

### 3.6. Enzyme Assays

#### 3.6.1. Laccase Activity

The activity of each laccase was determined with ABTS as the substrate. The change in the absorbance was recorded at 420 nm using a Biotek Eon 2 Microplate reader (Biotek, Winooski, VT, USA) at 30 °C. In brief, 15 µL of enzyme solution were mixed with 0.5 mM substrate in 50 mM phosphate buffer at pH 3.0 in a total volume of 300 µL. The change in the absorbance was monitored over ten minutes. One unit of enzyme activity was defined as 1 µmol of substrate (ɛ = 36,000 L·mol^−1^·cm^−1^) oxidized per minute under the experimental conditions [20].

#### 3.6.2. Esterase Activity

Esterase activity was assayed using *p*-nitrophenyl butanoate as the substrate and monitoring the change in absorbance at 410 nm (15,000 L·mol^−1^·cm^−1^, pH 8.0) over ten minutes with a Biotek Eon 2 Microplate reader (Biotek, Winooski, VT, USA) at 37 °C. Twenty microliters of sample were mixed with 175 μL of 100 mM sodium phosphate buffer (pH 6.0) and 5 μL of 50 mM *p*-nitrophenyl butanoate in ethanol. The increase of absorbance was monitored at 37 °C at 410 nm for 20 min. One unit of enzyme activity was defined as the release of 1 μmol nitrophenol per minute under the specified conditions [21].

#### 3.6.3. Lipase Activity

This assay was performed by measuring the increase in absorbance at 410 nm produced by *p*-nitrophenol released from 0.4 mM *p*-nitrophenyl butanoate in sodium phosphate buffer (50 mM, pH 7.0) at 37 °C. To start the reaction, the lipase solution or suspension (20 µL) was added to the substrate solution (175 µL buffer, 5 µL *p*-nitrophenyl butanoate). One international unit of activity was defined as the amount of enzyme that hydrolyzed one µmol of *p*-nitrophenol butanoate per minute under the conditions [22]. All enzyme assays were performed in duplicate, and the standard deviation was found below 5%.

### 3.7. Curcumin Transformation

#### 3.7.1. Chemical Acetylation of Curcumin

Acetylated curcumins were synthesized chemically as reference compounds using acetic anhydride. One mmol of curcumin was dissolved in 150 mL ethyl acetate and mixed for 20 min. After dissolving was completed, four mmol acetic anhydride were carefully added. After six hours, the reaction was stopped by adding one drop H_2_O_2_. Reaction yield (consumption of curcumin) and product identification were carried out by LC-MS.

#### 3.7.2. Lipase-Catalyzed Acetylation of Curcumin

Before each experiment, ethyl acetate and toluene as the solvent and the acyl donor (vinyl or geranyl acetate) were stored over Na_2_SO_4_. The reaction was carried out in 2 mL ethyl acetate and toluene (10:90) in sealed 30-mL glass vials at 40 °C with continuous stirring using a glass magnetic stir bar (150 rpm). The powdered CAL was added to a final concentration of five mg·mL^−1^. Sodium acetate buffer 50 mM, pH 6, was added at 4% to the reaction solution. Over the incubation, time samples were taken, filtered using 0.45-µm filter (Chromafil PET-45/25, Macherey-Nagel) and then analyzed directly by LC-MS.

#### 3.7.3. Monoacetyl Curcumin Degradation

The two-phase reaction system was made up of 2.0 mL of 0.5 mM monoacetyl curcumin (concentration calculated according to a curcumin standard) together with a minor impurity of diacetyl curcumin (concentration not affected by the laccase present) in 2.0 mL diethyl ether and 2.0 mL respective enzyme solution (1.19 U·mL^−1^, ABTS-assay, 30 °C, pH 3) in 50 mM sodium phosphate buffer, pH 5.5, under continuous vortexing at 1300 rpm (Heidolph, Germany) for 20 h at room temperature. A control sample with the buffer, but without enzyme, was treated under the same conditions. After separation of the diethyl ether phase, the aqueous buffer was re-extracted three times with 2.0 mL diethyl ether, and the combined organic phases were dried over night with Na_2_SO_4_. The degradation rate of acetyl curcumin was determined by LC-MS, and volatile degradation products, such as vanillin acetate, were analyzed by GC-MS.

#### 3.7.4. Esterase-Catalyzed Deacetylation of Vanillin Acetate

Three esterases were used for the hydrolysis of vanillin acetate. The hydrolysis was carried out in a glass vial containing 2 mL of 1 mM vanillin acetate dissolved in diethyl ether/hexane and 2 mL sodium phosphate buffer 50 mM, pH 5 and 6.5. The reactions were initiated by adding enzyme solution with an activity of one U·mL^−1^ to the reaction mixture and placed in a vortex shaker at 37 °C for five hours. The samples were extracted three times with diethyl ether, and the combined fractions were dried over sodium sulfate and analyzed by GC-MS using the external standard, 3,4-dimethoxybenzaldehyde (final concentration 125 mg·L^−1^). The reproducibility of three repeated transformations showed a relative standard deviation of typically 3%.

### 3.8. Gas Chromatography

#### 3.8.1. Gas Chromatography/Flame Ionization Detection

For each sample, 1 μL was injected on-column in an Agilent 7890A gas chromatograph (Agilent, Waldbronn, Germany) equipped with a cool on-column injection port and a 30 m × 0.32 mm i. d. × 0.25 μm CP-Wax 52 CB column (Varian, Darmstadt, Germany). The oven temperature program was 40 ° C for 3 min, raised at 3 °C per minute to 230 °C and held for 10 min. Hydrogen was used as the carrier gas at a flow rate of 2 mL per minute. Quantification was carried out according to the external standard (3,4-dimethoxybenzaldehyde).

#### 3.8.2. Gas Chromatography/Mass Spectrometry

Gas chromatography-mass spectrometry (GC-MS) was conducted using a GC 8000 coupled to an MD 800 mass-selective detector (Fisons, Mainz-Kastel, Germany) equipped with a cool-on-column injection port and a 30 m × 0.32 mm i.d. × 0.25 μm CP-Wax 52 CB column (Varian). The samples were injected using the same oven program as for GC/FID, but helium at a flow rate of 1.2 mL per minute was the carrier gas. Mass spectra were acquired using electron impact ionization at 70 eV and a 200 °C source temperature. Reaction products were identified by comparing their RIs (Resonance-ionization) and mass spectra with those of authentic standards.

### 3.9. Liquid Chromatography/Mass Spectrometry

For the identification of vanillin, curcumin and acetylated curcumins, as well as for the determination of the molar mass of the expected oxidation/polymerization products thereof, high performance liquid chromatography coupled to a triple quadrupole mass analyzer was used (Varian 212 LC pump, Pro Star 325 UV-Vis detector, 320 TQ-MS mass spectrometer). The MS was conducted simultaneously in the ESI positive and negative mode with a scan range of *m/z* 110–500 or *m/z* 300–1200, respectively. 

The MS parameters for ESI(+)/ESI(−) were: capillary voltage +30 V/−40 V, needle voltage 5000 V/−4500 V, nebulizer gas (N_2_) 379 kPa, drying gas 207 kPa at 350 °C. For HPLC, water and acetonitrile (MS-grade), both containing 0.1% formic acid, were used as the mobile phase, and the following linear gradient was used: 10% acetonitrile for three minutes, up to 90% acetonitrile within 20 min, hold for five minutes and back to start conditions. The separation was performed on an RP-18 HD column (Eurosphere 100-C18-5-HD, 250 × 4 mm, 5 µm, Macherey-Nagel) at a flow rate of 0.3 mL per minute. Additionally, UV absorption was monitored at 280 and 425 nm.

## 4. Conclusions

During the coming years, the flavor market is expected to increase, and biotechnology will contribute to guaranteeing the supply [1,23]. This work showed that laccases are suitable for the oxidative cleavage of acetyl curcumin in a cofactor- and mediator-independent reaction. A three-enzyme system provided protection/deprotection chemistry, with the selective acetylation of one of the two phenolic hydroxyl groups of curcumin as the key step. As a result, one phenol moiety of the symmetric molecule was protected against attack of the laccase, while the other vanillin moiety was inevitably lost to oligomerization.

Laccase-catalyzed reactions are governed by the structure of the phenolic substrate, the redox potential of the enzyme (high, middle or low), the presence and choice of a mediator and the usual parameters, such as reaction pH, temperature and solvent composition [24]. A refined system, including a food-grade mediator, might convert both acetyl vanillin moieties of an enzymatically-synthesized diacetyl curcumin into ‘natural’ vanillin. To this end, the first enzymatic step must be made more efficient by using a lipase able to accept both curcumin and monoacetyl curcumin as substrates. Some representatives out of the large set of lignolytic enzymes of higher fungi might be even more suitable for the acetylation, cleavage and hydrolysis of phenolic substrate molecules to yield ‘natural’ flavor compounds.
